# Phenotypic screening in Organ-on-a-Chip systems: a 1537 kinase inhibitor library screen on a 3D angiogenesis assay

**DOI:** 10.1007/s10456-023-09888-3

**Published:** 2023-07-26

**Authors:** Camilla Soragni, Karla Queiroz, Chee Ping Ng, Arthur Stok, Thomas Olivier, Dora Tzagkaraki, Jeroen Heijmans, Johnny Suijker, Sander P. M. de Ruiter, Aleksandra Olczyk, Marleen Bokkers, Frederik Schavemaker, Sebastian J. Trietsch, Henriëtte L. Lanz, Paul Vulto, Jos Joore

**Affiliations:** 1 MIMETAS BV, De Limes 7, 2342 DH Oegstgeest, The Netherlands; 2 Department of Cardiology, Maastricht University, Maastricht, The Netherlands

**Keywords:** High throughput screening, Angiogenesis, Protein kinase inhibitors, Phenotypic screening, Organ-on-a-Chip, Microphysiological systems

## Abstract

Modern drug development increasingly requires comprehensive models that can be utilized in the earliest stages of compound and target discovery. Here we report a phenotypic screening exercise in a high-throughput Organ-on-a-Chip setup. We assessed the inhibitory effect of 1537 protein kinase inhibitors in an angiogenesis assay. Over 4000 micro-vessels were grown under perfusion flow in microfluidic chips, exposed to a cocktail of pro-angiogenic factors and subsequently exposed to the respective kinase inhibitors. Efficacy of compounds was evaluated by reduced angiogenic sprouting, whereas reduced integrity of the main micro-vessel was taken as a measure for toxicity. The screen yielded 53 hits with high anti-angiogenicity and low toxicity, of which 44 were previously unassociated with angiogenic pathways. This study demonstrates that Organ-on-a-Chip models can be screened in high numbers to identify novel compounds and targets. This will ultimately reduce bias in early-stage drug development and increases probability to identify first in class compounds and targets for today’s intractable diseases.

**Supplementary Information:**

The online version contains supplementary material available at 10.1007/s10456-023-09888-3.

## Introduction

The discovery and development of novel drugs are among the most complex and costly processes known to mankind. A typical trajectory lasts 12 years and costs up to $3bn [1]. In drug discovery, high-throughput molecular screens have proven to be an efficient means to identify inhibitors of specific protein activities or simple biological processes. However, the translation of these compounds to efficacious therapies has proven itself much less efficient, contributing to current clinical failure rates exceeding 90%, in particular for chronic, high prevalence disease [2]. One of the underlying causes is the lack of human, biologically comprehensive, predictive assays throughout drug development. In particular, during compound screening and identification, assays are the simplest, due to the lack of comprehensive assays with sufficient throughput. We hypothesize that overly reductionistic approaches in the early stages of drug discovery are an important contributor to compound failure during clinical stages [3]. Consequently, the availability of biologically more comprehensive assays in these early stages may lead to lower attrition in the clinic.

In attempts to lower drug attrition rates, recent years saw a trend to improve the translatability of cell-based assays aiming to improve decision making in all phases of drug research and increase chances of successful drug development. 3D tissue culture, in which cells are embedded in an extracellular matrix (ECM) or clustered in spheroids have been shown useful in various stages of drug research [4, 5], including screening with multicellular tumor spheroids (MTS) of colon cancer cells to identify compounds with anticancer activity [6] or which target specifically the inner core of MTS [7]. Organoid technology from pluripotent or adult stem cells have been shown to differentiate into various lineages of cell types of the respective organ [8]. These organoids have been utilized in screens to identify epithelial-mesenchymal transition-reversing drugs from an epigenetic drug library as new therapeutics for breast cancer with organoids from claudin-low mammary tumors [9]. Patient-derived organoids of colorectal cancer were applied in a functional antibody screening to select a bispecific antibody [10]. Furthermore, kidney organoids derived from healthy, Wilms or malignant rhabdoid tumor (MRT) were used to identify drugs for MRT [11].

Organ-on-a-Chip technology aims to recapitulate human physiological processes by combining cell culture and microengineering techniques in miniaturized cell cultures inside microfluidic chips. Aspects such as perfusion flow, layered tissue architecture, mechanical strain, precise control over gradients and improved handling are added to the toolbox of 3D cell culture [12]. These chips typically emphasize technological complexity over throughput [13] and are therefore primarily being used in preclinical evaluation, rather than discovery, of drug candidates where multi-Organs-on-a-Chip models are used for toxicological study with heart-muscle-neuron-liver-on-a-chip [14] or to evaluate pharmacokinetics and pharmacodynamics of chemotherapeutic agent with a liver-tumor-marrow model [15] as well to study absorption, distribution, metabolism, and excretion (ADME) in a body-on-a-chip which comprises intestine, liver, non-vascularized liver, and blood–brain barrier [16]. The size and complexity of these devices including their peripheral equipment often limits the practical applicability for high numbers of assays needed for screening campaigns and compound confirmation.

Efforts towards integrating multiple chips into microtiter plate format devices [17, 18] have tremendously increased compatibility with standard lab equipment and scalability. A microtiter platform comprising 40 chips in parallel was utilized to assay over 357 chips comprising 3D perfused intestinal tubules and shown to yield highly reproducible results [19]. Ragelle and colleagues utilized this same platform to screen a small compound library on permeability in the context of retinal dysfunction [20]. Yet high-throughput screening of thousands of compounds needed for phenotypic target or compound identification remains to be demonstrated.

Here we report the first large scale phenotypic screen utilizing Organ-on-a-Chip technology. We determined the anti-angiogenic potential of a library of 1537 protein kinase inhibitors. The screen utilized a novel plate format comprising 64 microfluidic chips underneath a microtiter plate. Previously reported protocols for growing 3D ECM supported micro-vessels and subsequent angiogenesis induction [21] are adapted to fit this format. We utilized automated liquid handling and image analysis to enable phenotypic screening of the compound library in more than 4000 chips. Efficacy and toxicity were evaluated simultaneously by monitoring both sprouting and integrity of the main micro-vessel. Hit compounds were mapped against pathways and a dose response study was performed for two selected candidates. This approach exemplified how state-of-the-art cell culture technology, such as Organ-on-a-Chip-based models, can be utilized for the discovery of novel targets and compounds in a phenotypic screening.

## Results

### Plate and assay setup

**Fig. 1:**
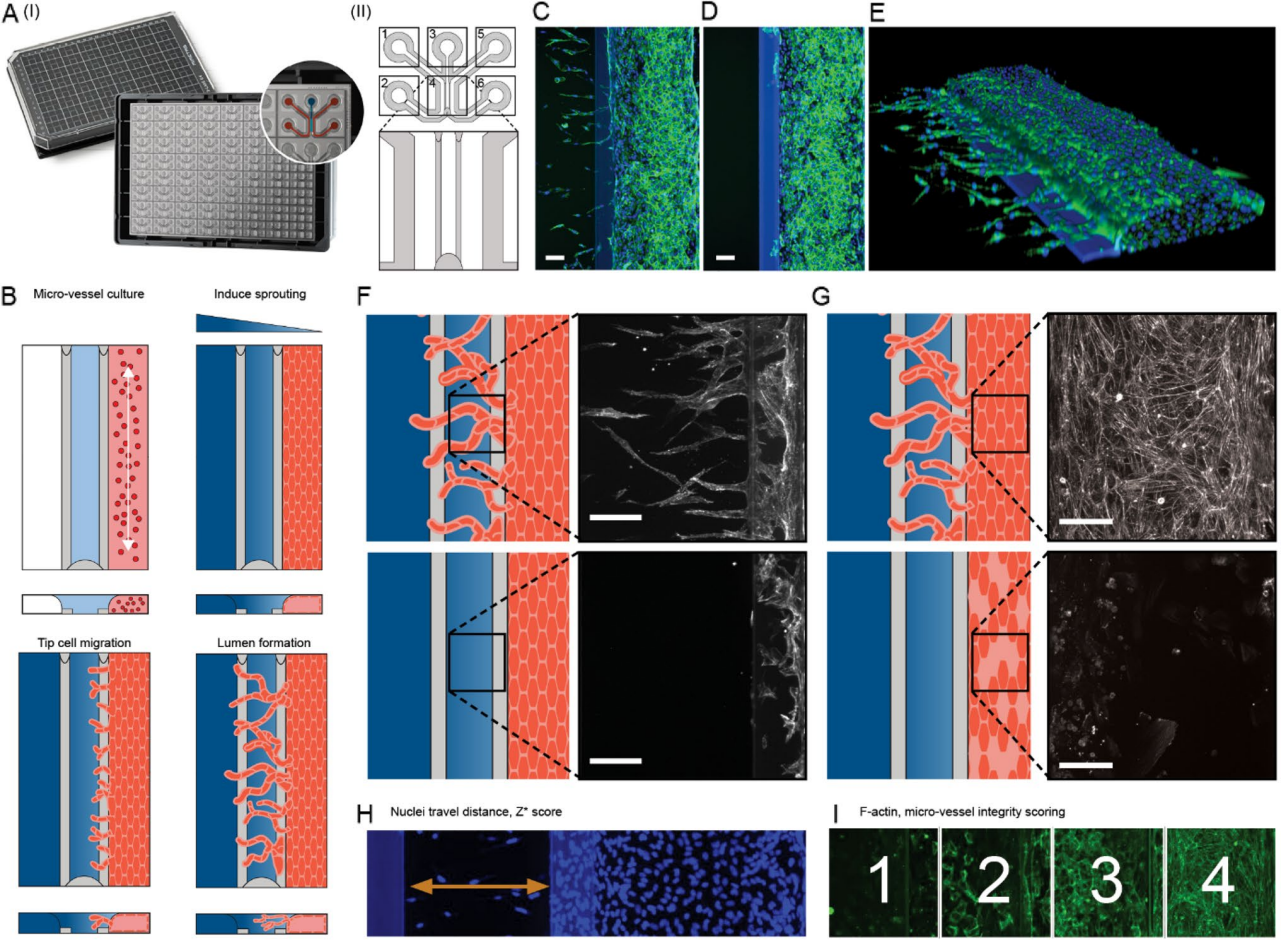
Angiogenesis assay: **A** (I) OrganoPlate 3-lane 64 comprising of 64 chips underneath a microtiter plate (II) Schematic of a single chip, comprising 3-lanes; **B** schematic of angiogenesis assays, comprising steps of gel loading, cell seeding, formation of a micro-vessel against the ECM gel (micro-vessel culture). A gradient of angiogenic factors (induce sprouting) induced sprouts formation (tip cell migration and lumen formation). Library exposure was performed in parallel **C** Representative max projection images of a confocal Z-stack of vehicle control **D** unstimulated, and **E** 3D reconstruction of the vehicle control (blue: DNA, green: F-actin); **F** inhibition of angiogenesis was measured in the gel region and **G** toxicity was assessed by micro-vessel morphology. **H** Inhibition was quantified by maximum travel distance of nuclei; **I** toxicity was quantified by scoring morphology following F-actin staining (green) into four categories. Scale bars in the images in **C**, **D**, **F** and **G** are 100 µm

Figure 1A shows the OrganoPlate 3-lane 64. The platform was based on a microtiter plate footprint and comprises on its flipside 64 microfluidic chips. Chips were spaced at 8 mm pitch, such that inlets of similar function could be addressed by multichannel pipettes as utilized in standard lab automation equipment (Fig. S1). A chip comprised of three microfluidic lanes. The central lane was filled with a collagen gel precursor. The gel precursor was spatially confined by two Phaseguides [22] and allowed to gelate. Endothelial cells were seeded subsequently to gelation, the plate was placed on an interval rocker and cells were allowed to form a 3D tubule upon application of perfusion. The tubule (micro-vessel) was thereafter exposed to a cocktail of pro-angiogenic factors inducing angiogenesis in addition to the compound under investigation (Fig. 1B). Figure 1C and D show a representative image of a sprouted and non-sprouted micro-vessel, as well as a 3D representation (Fig. 1E).

### A 1537 protein kinase anti-angiogenic compounds screen

1537 kinase inhibitors were added together with the cocktail of angiogenic factors to assess their inhibitory effect on angiogenic sprouting. In addition, we included three different controls: (1) 528 vehicle control chips (vessels with sprout cocktail without inhibitor), (2) 264 chips that were exposed with Sunitinib and (3) 264 chips that were unstimulated (vessels without sprout cocktail and inhibitor) (Fig. S2). Samples were screened in duplicate. The screen was split in four batches, for a total amount of 4130 chips and 65 plates. Liquid handling automation was employed for the steps of ECM loading, cell seeding, compounds addition, fixation, and staining. A quality control step was implemented post tubule formation prior to compound exposure, by visual inspection of the phase-contrast image of the micro-vessel with 96.5% (3987) passing QC. High replicability (Fig. S3) was observed for both unstimulated (*n* = 248, CV 14.1%) and vehicle control, (*n* = 494, CV = 12.1%). The assay had an excellent *Z*′ factor of 0.8 [23, 24].

**Fig. 2:**
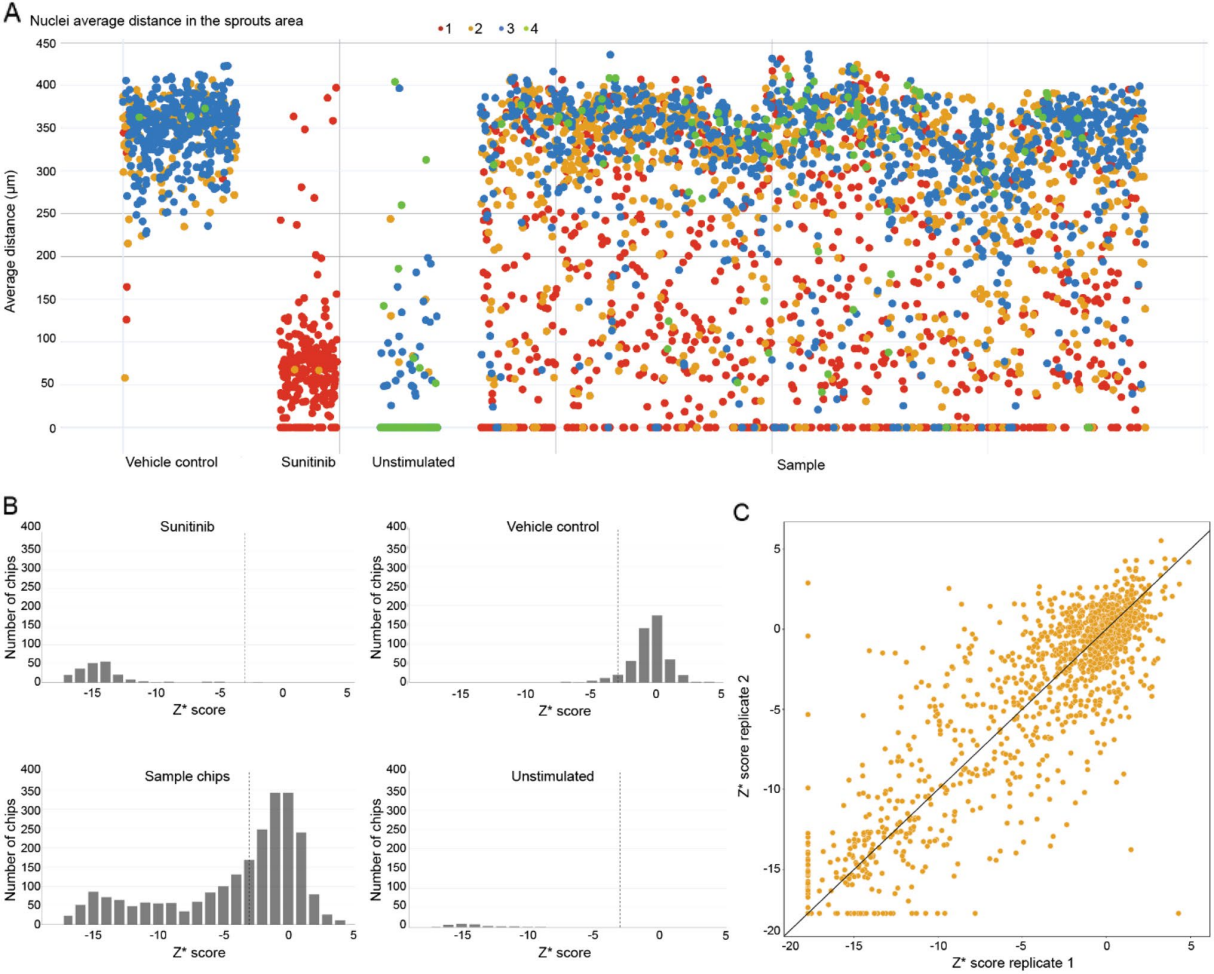
Screening of a 1537 kinase inhibitor library on the inhibition of angiogenesis. **A** Average distance of the ten furthest nuclei with respect to the micro-vessel in µm, each dot represents a chip and was color coded for toxicity as assessed by micro-vessel actin network integrity. A score of 4 being a fully intact cytoskeleton network, while 1 being fully degraded micro-vessel; **B** Distribution of chips by Z* score based on the average distance per condition; Dashed line indicated cut-off for inhibition classification (Z* score = − 3). **C** Correlation of Z* scores between sample replicates with the *y* = *x* trendline

Following compound and cocktail exposure, chips were fixated and stained for actin and nuclei. Images were acquired through high content image microscopy. The maximum travel distance of nuclei was used as a measure for sprouting length (Fig. 1E, H), while the quality of the tubes was scored on a 4-points scale based on actin structure (Fig. 1G, I). Vehicle-control-exposed vessels showed a median sprouting distance of 352.1 µm while Sunitinib-exposed vessels sprouted a median distance of 62.2 µm. Sample chips were distributed in between these values with a median sprouting distance of 294.3 µm (*n* = 2906) (Fig. 2A). From the nuclei distance we quantified the robust Z* score (Fig. 2B) and found a good separation between Z* scores of unstimulated and vehicle controls. The Spearman correlation between duplicates (Fig. 2C) was 0.84. Different ranges of robust Z* scores were defined in association with no (above − 3), mild (− 3 to − 9), moderate (− 9 to − 15), and high (below − 15) levels of inhibition. Representative images per inhibition levels (ILs) are shown in Fig. S4.

Most vehicle control assays (96.0%) showed no inhibition, while Sunitinib was mainly distributed between moderate (54.8%) and high (42.5%) inhibition. Samples showed a wide range of inhibition levels with no, mild, moderate, and high inhibition observed in 52.1%, 15.5%, 12.8%, and 19.7% of chips, respectively (Table S1).

Due to our model’s configuration in a single horizontal plane, we could evaluate the effect of compounds on sprout inhibition and also on the tubule. Toxicity on the tubule was assessed by expert visual scoring of its actin network integrity. We classified into four ordinal categories of intactness representing no, minor, medium, or major toxicity (scores of 1–4 respectively, see Fig. S5 for representative images). Only 3.5% of chips showed no toxicity, while minor, medium, and major toxicity was observed in 38.3%, 25.3%, and 32.9% of chips, respectively. For control chips the majority of vehicle (74.7%) and unstimulated datapoints (75.4%) showed fully healthy tubes while 99.6% of Sunitinib showed signs of toxicity. Most compounds showed at least some toxicity, with only 3.1% (90 chips) showing no toxicity at all. We observed minor, medium, or major toxicity in 32.2% (937 chips), 29.3% (852 chips), and 35.3% (1027 chips), respectively. Replicability was high with 97.4% of duplicates showing the same (67.0%) or adjacent (30.4%) integrity scores.

### Screen analysis and hit selection

**Fig. 3:**
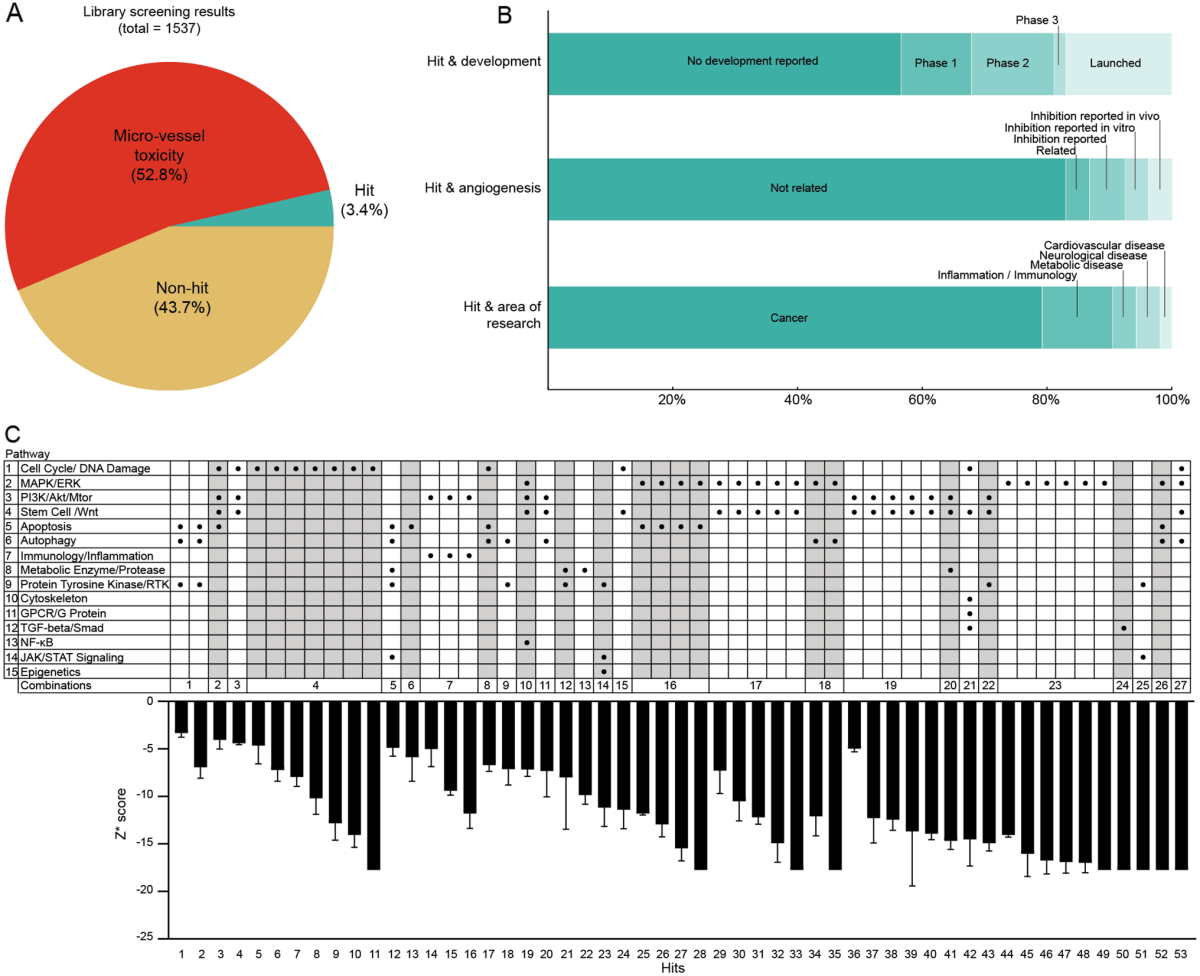
Data and pathway analysis: **A** pie chart showing percentage of hits, non-hits, and compounds with micro-vessel toxicity; **B** analysis of hit compounds in relation to clinical focus; **C** target pathway analysis of hits: main pathways and combination of main pathways in relation to their Z* scores [expressed as average ± standard deviation (AVG ± STD)] per replicate of hits. Gray shading highlighting combinations of pathways with multiple hit compounds

Out of 1537 compounds screened we identified 53 hits (3.4%) (Fig. 3A). Within the non-hits (1484), the majority was excluded (812, 52.8%) due to micro-vessel toxicity quantified with integrity scores 1 and 2 as detrimental effects on the main micro-vessels were observed.

Majority of the hits (56.6%) were compounds with no development reported (Fig. 3B) and thus might offer novel applications for these compounds. The remaining compounds were in various stages of clinical development with 11.3%, 13.2%, and 1.9% being in phase 1, 2, or 3, clinical trials, respectively, and 17.0% being launched. Nine hits were previously associated with anti-angiogenic properties: Brivanib (Z* score = − 7.2 ± 1.7), PD0325901 (Z* score = − 17.9 ± 0.0), Butein (Z* score = − 4.9 ± 0.9), Theaflavin-3,3′-digallate (TF3) (Z* score = − 7.2 ± 0.7), GSK-872 (Z* score = − 5.9 ± 2.6), AZ-628 (Z* score = − 11.9 ± 0.2). Within this category, five compounds (9.4% of hits) were already claimed to be directly involved or related to angiogenesis, four compounds (7.5% of the hits) already showed angiogenic inhibition in other cell-based or in vivo models, while 44 compounds (83% of the hits) had not been previously associated with angiogenesis (Fig. 3B). In terms of research area, 79.2% of hits were associated with cancer, 11.3% in inflammation/immunology, 3.8% in neurological and metabolic disease, and 1.9% in cardiovascular disease research.

According to the annotation of the library used, the compounds were associated with 15 main pathways organized in 27 combinations. Figure 3C shows the Z* scores of hit compounds and their relation to pathways and pathway combinations. 20 hit compounds (37.7%) were associated with the MAPK/ERK pathway, and 19 hits compounds (35.8%) were associated with the Wnt pathway. Furthermore 14 hit compounds (26.4%), 13 hit compounds (24.5%), 11 hit compounds (20.8%), and 10 hit compounds (18.9%) were associated with PIK3/Akt/mTOR, Cell Cycle/DNA Damage, Apoptosis, and Autophagy pathways, respectively. Hit compounds acted on up to 5 of the main pathways simultaneously. Out of 27 combinations of main pathways 8 were common between multiple hit compounds (combination number 1, 4, 7, 16, 17, 18, 19, 23 in Fig. 3C). The combinations which contain 4 or more hits (combination number 4, 16, 17, 19, 23) targeted 1 or 2 main pathways. For example, the combination number 23 of 6 hits targeted the MAP/ERK pathway. The MAPK/ERK or Cell cycle/DNA damage pathways were the most frequently targeted pathways within this combination of hits. MAPK/ERK alone or in combination with other pathways caused mainly moderate and strong inhibition.

### Morphological classification and target association

**Fig. 4:**
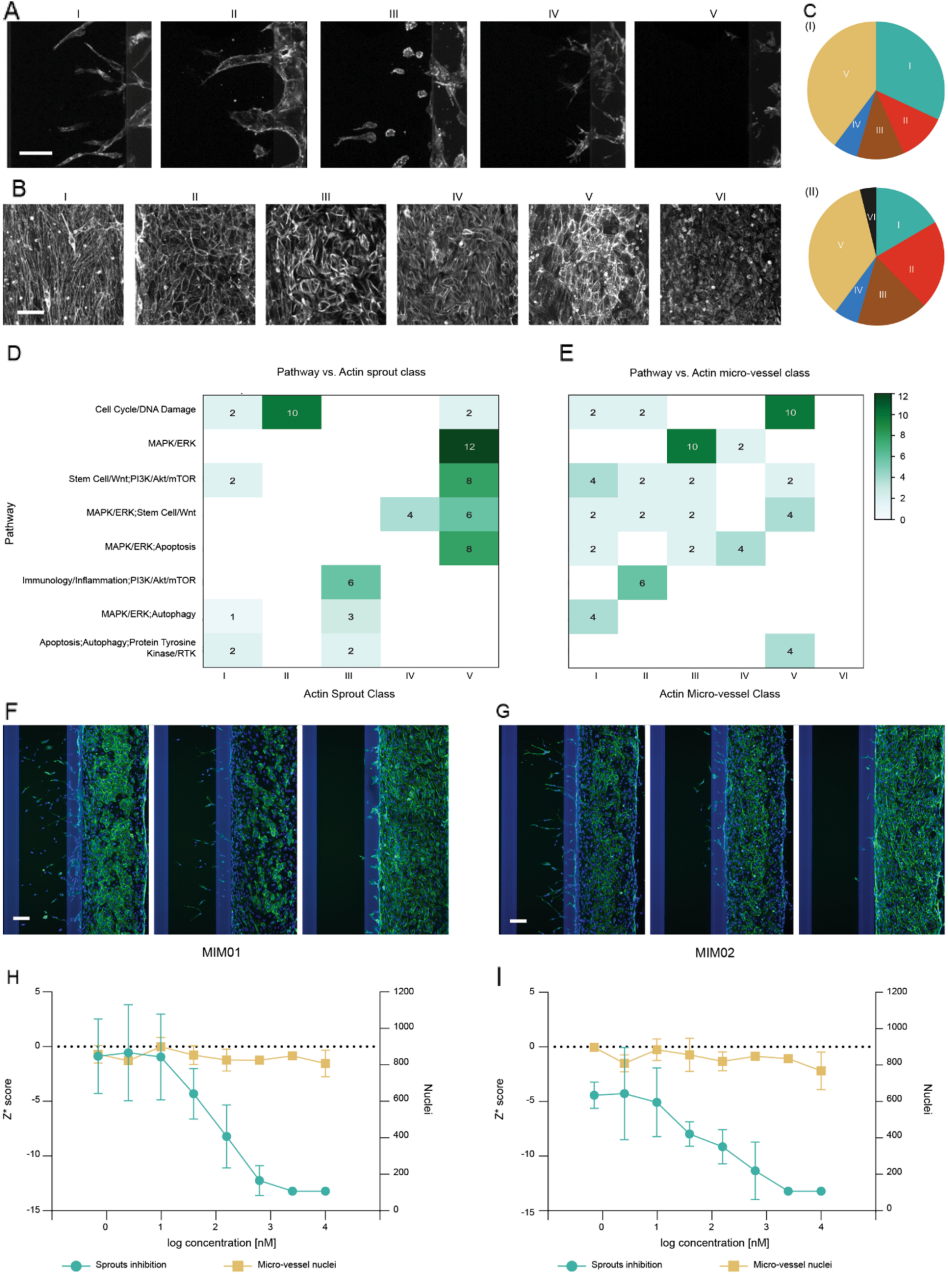
Morphological analysis of hit compounds. Representative images of F-actin staining classified in **A** five categories of sprout morphology and **B** six categories of tubule morphology identified by analyzing the 53 hits; **C** pie diagrams depicting proportion of hits per sprout class (i) and tubule class (ii); **D**, **E** heatmaps showing the most common pathway combinations for sprout and tube classes, respectively, from analysis of hit clusters (i.e., pathway combinations affected by multiple hit compounds; this comprised of 34 hits (68 data points) from the combination numbers 1, 4, 7, 16, 17, 18, 19, and 23 in Fig. 3C); **F**, **G** dose–response cellular images from chips exposed to hit compounds MIM01 and MIM02 (from the MAPK/ERK cluster; combination number 23 in Fig. 3C) from left to right at lowest (0.7 nM), middle (39 nM), and highest concentrations (10 µM), with F-actin in green and nuclei in blue; **H**, **I** dose–response curves for MIM01 and MIM02, respectively, with Z* scores on the left primary *Y*-axis and nuclei count in the micro-vessel on the right secondary *Y*-axis. Data were expressed as AVG ± STD. Scale bars in **A**, **B**, **F** and **G** are 100 µm

We further analyzed the 53 hits by running a more detailed morphological analysis of the F-actin network organization in the micro-vessel and sprouts. Through visual assessment, three experts classified images based on similarities and found five general classes for sprouts (Fig. 4A) and six classes for vessel morphology (Fig. 4B). For sprouts, class I (“regular”) corresponded with the morphology typical of the vehicle control, class II (“broad”) was reserved for sprouts with a broad diameter, class III (“round") comprised single rounded or dead cells, in class IV (“branched”) and V (“no sprouts”) showed the highest inhibition with almost no sprouts or very small and spread sprouts. In assessing the tubule morphology, class I (“elongated”) referred to a fibroblast like network morphology, which might indicate a differentiation effect of the compound; class II (“regular”) referred to a well-formed and intact network; class III (“stressed”) were stressed organized actin fibers which could indicate a higher degree of polarization in response to compounds; class IV (“highly stressed”) were stressed unorganized actin fibers, which we hypothesized to be an evolution of class 3; class V (“heterogeneous”) showed heterogeneous morphology and class VI (“no actin network”) showed a strongly affected actin network, which was found in just 1 hit (for both data points). “no sprouts” (41.5%) and “regular” (31.1%) were the most common morphology for sprouts (Fig. 4C(I)) while the most common tubule morphologies were “heterogeneous” (35.8%) followed by “regular” (20.8%), “elongated” (17.0%), and “stressed” (17.0%) (Fig. 4C(II)).

The morphological results were matched against pathways or pathway combinations (Fig. 4D, E) affected by multiple hit compounds (combinations number 1, 4, 7, 16, 17, 18, 19, and 23 in Fig. 3C). Hits associated with Cell Cycle/DNA damage showed mainly “broad” sprouts (10/14 Fig. 3D) and “Heterogeneous” tubules (10/14 Fig. 4E). Hits associated with the MAPK/ERK pathway presented “no sprouts” (12/12) and “Stressed” (10/12) or “Highly Stressed” (2/12) tubules, when in combination with autophagy sprouts showed a “round" morphology (3/4) and tubule were “elongated” (4/4). Images in Fig. S6 depicted all the hits and their duplicates affecting the MAPK/ERK pathway.

### Dose response testing

Two of the MAPK/ERK pathway affected hits (designated MIM01 and MIM02) were selected for dose response testing (Fig. 4F, G). An 8-points dose response study was executed utilizing the same assay as for the primary screen, but here we used nuclei count as indication of toxicity. We found an IC50 value of 100.0 nM for MIM01 (Fig. 4H) and 102.2 nM for MIM02 (Fig. 4I). Both compounds showed stable nuclei count in the micro-vessel at all concentrations, combined with a dose dependent inhibition of angiogenesis, suggesting that these were both safe and efficacious anti-angiogenic compounds. To obtain more insight in the effects of MIM01 and MIM02 on sprout formation, we further analyzed the data obtained from cultures exposed to lower compound concentrations (0–156 nM MIM01 or MIM02) as sprouts were absent in the higher concentration range. In these cultures, we observed that tip cells are present, and sprouts are initiated. However, sprout extension and lumenization seem to be impaired, which are both dependent on the proliferation of stalk cells [1, 2]. These results indicate that MIM01 and MIM02 both act on stalk cell proliferation. In addition, upon compound exposure, endothelial cells still migrate into the ECM and elongate, suggesting normal migratory behavior. Nuclei in MIM01 and MIM02-treated cultures appear bigger and more elongated compared to vehicle control (supplementary Fig. S7). MIM01 exposure resulting in larger nuclear size with similar shape regularity in comparison to vehicle controls might indicate cell senescence. By contrast, MIM02 induced larger nuclear size and more shape irregularity, suggesting nuclear damage [25].

## Discussion

The current study marks a step change in the use and scalability of Organ-on-a-Chip models in phenotypic screens. Incorporating more physiologically relevant models in screens will improve the identification of more effective compounds and potentially reduce failure in later developmental phases. As a first step in this direction, we report the application of a scalable Organ-on-a-Chip platform to perform a high-throughput phenotypic screen in combination with high resolution morphological analyses of an angiogenesis assay. This assay was composed of endothelial cells grown as micro-vessels against ECM and cultured under perfusion; and recapitulates major steps of in vivo angiogenesis, such as tip cell formation, cell migration, apical/basal polarity. The micro-vessels generated perfusable sprouts in response to a gradient of pro-angiogenic factors [21]. It is important to note that the conventional 3D assays commonly used, such as tube formation, endothelial cell-coated beads, and spheroid sprouting assays, often lack many of the essential elements present in our assay. The Organ-on-a-Chip platform we employed incorporates these crucial features, allowing for a more accurate and representative study of angiogenesis.

We further demonstrated the feasibility of scaling and applying our angiogenesis assay in a high-throughput screening setting. This assay was used to identify safe and efficacious anti-angiogenic compounds from an annotated protein kinase inhibitors library of 1537 compounds. Inhibitors that effectively reduced sprouts formation and exhibited a very limited impact on the integrity of the micro-vessel were considered hits. This selection was supported by the quantification of the average sprout length and changes in F-actin network organization of the micro-vessel. Having high-content imaging data on both the source micro-vessel as well as the angiogenic sprouts enabled us to combine accurate measurements of sprout inhibition and detailed visualization of morphological changes and toxicity.

Despite the complexity of the model, it was replicable and performed reliably as shown by the *Z*′ factors. Quality control, prior to sprouting mix and library exposure, showed consistency in establishing micro-vessels. After exposure the nuclei count in the micro-vessel of vehicle and unstimulated was also consistent across all the chips. The angiogenesis assay was shown to be robust and scalable enabling the identification of novel compounds acting on angiogenesis through previously unknown targets. The screen was performed at a high concentration (10 µM), emphasizing selective compounds which specifically target only sprouts and not the micro-vessel. This is especially valid for a compound class like protein kinase inhibitors which are notoriously promiscuous.

To evaluate vasculature related adverse effects of screened compounds, we assessed the cytoskeletal reorganization in the micro-vessels. The resulting morphological analysis enabled monitoring of much more subtle changes in cell phenotype than simple viability or proliferation. Furthermore, the detailed phenotype observed could be associated with specific affected pathways. Among the 53 hits identified the majority (20 hits) acted on MAP/ERK pathway, which in several studies had been shown to support angiogenesis. The strong inhibition in sprout formation or elongation observed in response to compounds targeting the MAP-kinase pathway indicated that pro-angiogenic mediators used in our model converge at this pathway likely at MEK level [26]. As a result, inhibition of MEK and ERK suppressed downstream signals essential for angiogenesis. On the other hand, most protein tyrosine kinase/RTK inhibitors included in the screen (256 in total) were not considered hits as these not only inhibited sprouts formation/elongation, but also affected micro-vessel integrity (129, 50.4%).

The selected hits included nine compounds which were already associated with angiogenesis, which supports the suitability of the method to identify anti-angiogenic compounds. Brivanib is a FGFR and VEGFR inhibitor currently in phase 3 of clinical development. In preclinical studies this compound induced tumor stasis and extended overall survival in a mouse model of pancreatic neuroendocrine tumor [27]. PD0325901 inhibits MEK, ERK phosphorylation, VEGF and IL-8 at transcriptional level. It was known to modulate cell cycle and apoptosis signaling at protein level and affect genes involved in the negative regulation of MAPK signaling [28]. Butein suppressed micro-vessel formation in vivo and VEGF-induced vessel sprouting, without inducing cytotoxicity [29]. Theaflavin-3,3′-digallate (TF3) inhibited tube formation in HUVEC and decreased microvascular density in vivo with a chick chorioallantoic membrane assay, and had also been hypothesized for strong anti-angiogenic cancer treatment [30]. In vivo testing of GSK-872, a catalytic receptor-interacting protein 3 (RIP3) inhibitor, showed its capability to suppress angiogenesis [31]. AZ-628, a pan-Raf kinase inhibitor, also inhibited activation of VEGFR2 and because of its similar cross reactivity to Sorafenib, it might be used as an anti-angiogenic agent as well [32]. The effect of these compounds was readily captured in our screen.

An example compound showing the need for early, biologically relevant, safety assessment as well as efficacy testing was Regorafenib. This compound made it into clinical trials based on classical preclinical models, but was later found to cause hypertension [33–35] and hepatotoxicity [36] in the clinic. In our screen, the compound showed major anti-angiogenic efficacy (Z* = − 17.7) but also showed significant toxicity (Medium toxicity). While this result was by no means a full validation of its predictivity, it suggested the potential of high-fidelity models for both efficacy and toxicity in preclinical screening.

In summary, this study was the largest Organ-on-a-Chip phenotypic screen performed thus far. We demonstrated that the angiogenesis assay in the OrganoPlate was reliable, robust, and ready to be applied in a high-throughput setting. We screened a protein kinase inhibitors library on an angiogenesis assay and developed a new hit selection approach. Our hits list consisted of safe and effective compounds that selectively interfered with sprouts formation and produced limited effects on the main endothelial micro-vessel. These findings were supported by the assessment of sprouts inhibition and integrity of micro-vessel cytoskeleton. Furthermore, we observed the clustering of hits which affected the same signal transduction pathways and induced similar morphological responses. Scalable Organ-on-a-Chip models are set to redefine phenotypic drug discovery, by leveraging high fidelity biological models, high-content imaging, and phenotypic analysis at an unprecedented scale.

## Materials and methods

### Cells and reagents

Primary Human Umbilical Vein Endothelial Cells (HUVECs) and EBM-2 medium supplemented with EGM-2 SingleQuots were purchased from Lonza. Rat Tail Collagen Type I was acquired from Corning, while the following reagents were from Sigma Aldrich, except indicated otherwise: Acetic Acid, HEPES (Lonza), Sodium Bicarbonate (NaHCO_3_), Hanks’ Balanced Salt Solution (HBSS; Gibco), Phosphate-Buffered Saline (PBS) (Gibco), Vascular Endothelial Growth Factor (VEGF rhVEGF-164; PeproTech), Sphingosine-1-phospate (S1P), Phorbol Myristate Acetate (PMA), Dimethylsulfoxide (DMSO), Sunitinib Malate, Formaldehyde, ActinGreen (Thermo Fisher Scientific), and Hoechst 33342 (Thermo Fisher Scientific). The compound library, of 1537 protein kinase inhibitors, was customized with and supplied by MedChemExpress, USA.

### Platform, instrumentation, and automation

The screening was performed semi-automatically using a pipetting robot (Biomek i5; Beckman Coulter) to mix and dispense the reagents, and a non-contact dispenser (MultiFlo FX; BioTek) to fix and stain the cultures. Manual work was only carried out to prepare reagents in a format suitable for the robotics and to move plates between instrumentations. It was possible to screen thousands of compounds in a single incubator using the OrganoPlate 3-lane 64 (Mimetas BV, Leiden, the Netherlands) (Fig. 1A(I)) and a pump-free rocking perfusion system (OrganoFlow).

Each OrganoPlate 3-lane 64 contained 64 microfluidic chips embedded at the bottom of a standard 384-well plate (Fig. 1A(II)). Each chip comprised of six wells in a 2 × 3 well grid; four of these wells were used as inlet and outlet for the perfusion channels [wells number 1–2 and 5–6 in Fig. 1A(II)], another well as inlet for the ECM channel [well number 3, Fig. 1A(II)], and the final well as the observation window [well number 4, Fig. 1A(II)] where the three microfluidic channels (400 × 220 µm, *w* × *h*) met (Fig. 1A(II)). In the observation window where cellular readouts were acquired, the central channel was the ECM channel while the perfusion channels were adjacent on the left and right. The channels were separated by two Phaseguides (100 × 55 µm, *w* × *h*) which enabled the retention of ECM through pinning; this allowed cultured cells grown in a perfusion channel to be in direct contact with the ECM in a barrier-free fashion. To achieve pump-free perfusion, we used the OrganoFlow to impose a physiologically relevant shear stress; this is a rocker platform which held the plate at a defined angle and enabled gravity force-driven perfusion induced by height differences between inlet and outlet of each channel through regularly flipping at a set time interval. Cells seeding and the assay were handled in an automated way to increase replicability between plates and batches.

### Vasculature model

The vasculature model was established as previously described [37, 38]. Rat tail collagen of final concentration 4 mg/mL was loaded into the chips with the automated liquid handler (Biomek i5), and incubated overnight at 37 °C. HUVECs were seeded by dispensing 1.25 µL of 8000 cells/µL in EGM-2 in the right perfusion channel, resulting in passive pumping as described previously [39] for a final concentration of 10 K cells/chip. The OrganoPlates were incubated for 2.5 h at a 75° angle to allow cell attachment before 50 µL EGM-2 medium was added to the outlets of the right perfusion channels. The plates were cultured on an OrganoFlow rocker rocking at an angle of 14° and an interval of 8 min for 72 h for proper tube formation. Phase-contrast images of formed micro-vessels were taken and used for quality control (QC) by expert visual assessment.

### Screening assay

The screen was performed in four batches. Samples were screened in duplicate for a total amount of 4130 chips and 65 plates used.

Chips used for compound exposure were referred in this paper as “Sample chips”. The compounds were screened at 10 µM (0.1% DMSO) concentration selected based on literature data [40–44] and at high concentration/potency to elucidate toxicity effects on the main vasculature. Compound additions were performed using the automated liquid handler with the compounds in EGM-2 added to the right perfusion channels containing the micro-vessels and compounds in an angiogenic cocktail mix of 50 ng/mL VEGF, 50 nM S1P, and 2 ng/mL PMA were added to left perfusion channel at 50 µL per well.

For controls, eight chips/plate of micro-vessels with sprouting mix and DMSO (vehicle control), four chips per plate treated with sprouting mix and Sunitinib inducing strong inhibition (Sunitinib), and four chips without sprouting mix and compounds (unstimulated) were used. Sunitinib was chosen because it was a well-known drug with anti-angiogenic effect [45]. After compound addition, the plates were cultured on the OrganoFlow for 48 h followed by fixation for 15 min using 3.7% formaldehyde in PBS and immunostaining for nuclei (5 µg/mL, Hoechst 33342) and F-actin (1× ActinGreen) using a MultiFlo FX washer.

### Image acquisition

Images were acquired by using ImageXPress XLS Micro HCI and ImageXPress XLS Micro Confocal (both from Molecular Devices). For phase-contrast image acquisition we employed a 4× 0.13 NA Plan Apo air objective (Nikon). For fluorescence we used a 10× 0.45 NA Plan Apo air objective (Nikon). For confocal data acquisition we utilized the following setup: A 0.45 NA Plan Apo air objective, a custom pre-image registration routine to ensure exact *X*/*Y* positioning of microfluidic features for every image, 100 ms exposure time for both fluorescent wavelengths (DAPI & FITC), a 60 μm Nipkow spinning disk configured for 220 µm of *Z*-height, with a Z-step increment of 3 µm.

### Screening data processing

We acquired phase-contrast images of micro-vessel before performing angiogenesis assay and epi-fluorescent images after the assay. All data was assessed for artifacts that might have occurred during image acquisition. The data processing was done through a combination of ImageJ/Fiji [46] and python pipelines. For the phase-contrast data we enhanced the brightness and contrast and created montage-views that correspond with the physical layout of the images on the OrganoPlate. These images, as previously mentioned, were used for quality control assessment by two experts during the analysis to exclude micro-vessels that were not well developed at the start of assay. Micro-vessels were scored from 1 to 4, with 1 indicating a severely disrupted tubule and score 4 a perfectly coherent and confluent tubule. Images which scored lower or equal to 2 were excluded.

The epi-fluorescent images were processed through multiple pipelines. One pipeline followed the same workflow as the phase-contrast data to create montage views that can be used for visualization and inspection. F-actin images were also rated manually from 1 to 4 by two experts. Images of F-actin, used to evaluate safety on micro-vessel, were scored based on the structure and integrity of the cytoskeleton which indicates the level of toxicity experienced in the micro-vessel, with one indicating major, two medium, three minor, and four no toxicity. Only chips of scores 3 and 4 were included for hit analysis. The second pipeline, applied on DAPI wavelength images where positions of the nuclei were extracted. For each image a background signal was approximated through a rolling ball algorithm which was subsequently subtracted. A feature enhancement routine through a Difference-of-Gaussians (DoG in short [47], algorithm enhanced the local S/N for every nucleus. The resulting image was converted into a binary mask through the application of an automatic threshold using the IsoData algorithm [48]. In the resulting binary image, touching objects were separated by applying a watershed algorithm [49]. The output of this routine was a binary mask of the identified nuclei, and the *X*/*Y* centroids and area of every identified nucleus.

Using the combined output of all three pipelines further data processing was done through a pipeline in python (supplementary information). From the *X*/*Y* centroids of each nucleus in the sprouts area we averaged the longitudinal location (*Y*) of the ten furthest nuclei. Data were normalized against the vehicle control and robust *Z* (Z*) score, an indication of the number of absolute median deviation (MAD) from the median of the vehicle control nuclei distance, was quantified [50]. Robust statistics were used since we have lower number of control replicates (4–8 chips) in our screening set-up per plate compared to typical high-throughput screens consisting of 96–384 wells (which could employ up to 16 wells of controls). Furthermore, robust statistics was less sensitive to outliers and more suited for non-parametric data [51, 52].

We used a python-pipeline to analyze QC, safety (micro-vessel integrity) and Z* scores to select properly developed micro-vessel, with a QC score greater or equal to 3, and compounds which did not affect micro-vessel integrity (F-actin staining), with an integrity score equal to or higher than 3, while they inhibited sprouts formation with a Z* score lower than − 3. Hits were selected based on the combinations of these three scores and the agreement between them in the duplicate.

### Target-pathway analysis

Based on annotations accompanying the library, and enrichment using public databases (Chembl, Pubchem [53, 54]), we further characterized the selected hits. For target- and pathway-dependent effect assessment, a pipeline in Python was developed.

The compound library contained information on all known associated targets and pathways. Most of the compounds had multiple associations on a target and pathway level. For all compounds, the multi-target/pathway associations were combined with the observed response within the screen. The frequency of unique combinations of pathways was counted and visualized next to the mean observed efficacy within the screen.

### Morphological analysis

F-actin and nuclei images of selected hits were further processed in ImageJ/Fiji to identify correlation between morphological changes and target affected. For this analysis, we split the images of the hits into two regions of interest (ROIs). The first region contained the endothelial micro-vessel, excluding the Phaseguide. The second region contained the sprouting vasculature, from the Phaseguide next to the micro-vessel until the other Phaseguide. The images were pre-processed in a similar manner for the initial screening analysis, correcting for artifacts and evening out the background signal over the images. Each ROI was scored by two experts based on the morphology of the micro-vessel or sprouts. Data was processed in Excel and graphs were generated in Graphpad and JMP statistical software.

### Dose–response

Vasculature models were established as previously described. We used the same angiogenesis assay and readout as for the primary screening with the only difference that we tested eight different concentrations, from 0.7 nM to 10 µM of the selected hits which affected MAP/ERK pathway. Each condition was tested in duplicate, and we used the same controls as for the primary screening. Images of nuclei were used to quantify the number nuclei in the micro-vessel and to extract position of the nuclei in the ECM area. Inhibition of sprouts was evaluated as before with the ten furthest nuclei and Z* scores. Data were handled and graphs were generated in Graphpad.

### Supplementary Information

Below is the link to the electronic supplementary material.

Supplementary file1 (DOCX 2859 KB)
