# Supplementary material for: Phenotypic screening in Organ-on-a-Chip systems: a 1537 kinase inhibitor library screen on a 3D angiogenesis assay
Source: Angiogenesis. 2023 Jul 26;27(1):37–49. doi: 10.1007/s10456-023-09888-3 (PMC10881651; doi:10.1007/s10456-023-09888-3)
Supplement: Supplementary file 1 — Supplementary file1 (DOCX 2859 KB) [file 10456_2023_9888_MOESM1_ESM.docx]

# Supplementary information


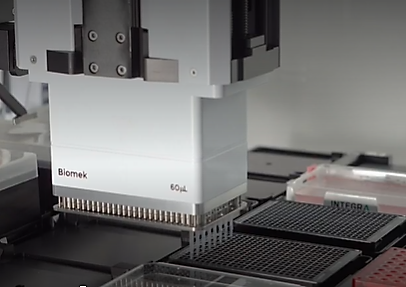


Fig. S 1 Automation friendly platform. The OrganoPlate is compatible with industry standard screening equipment such as automated liquid handlers.


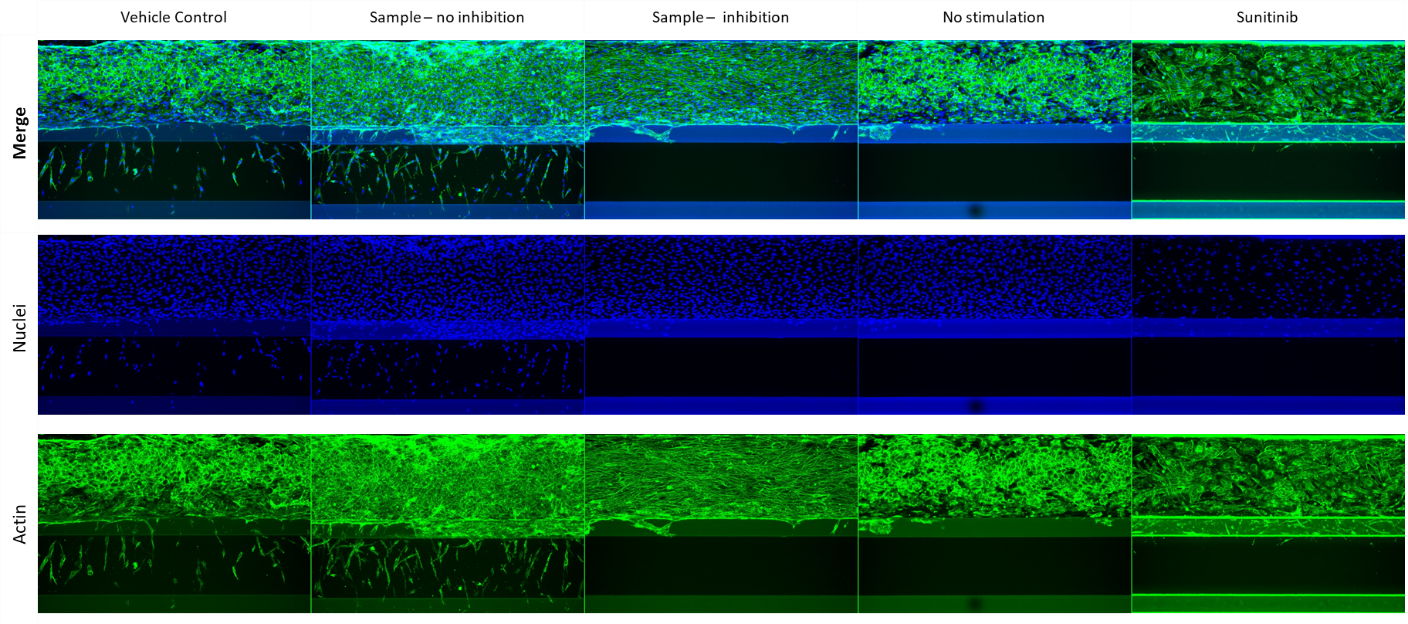


Fig. S 2 Representative images of controls and sample showing inhibition or no-inhibition.


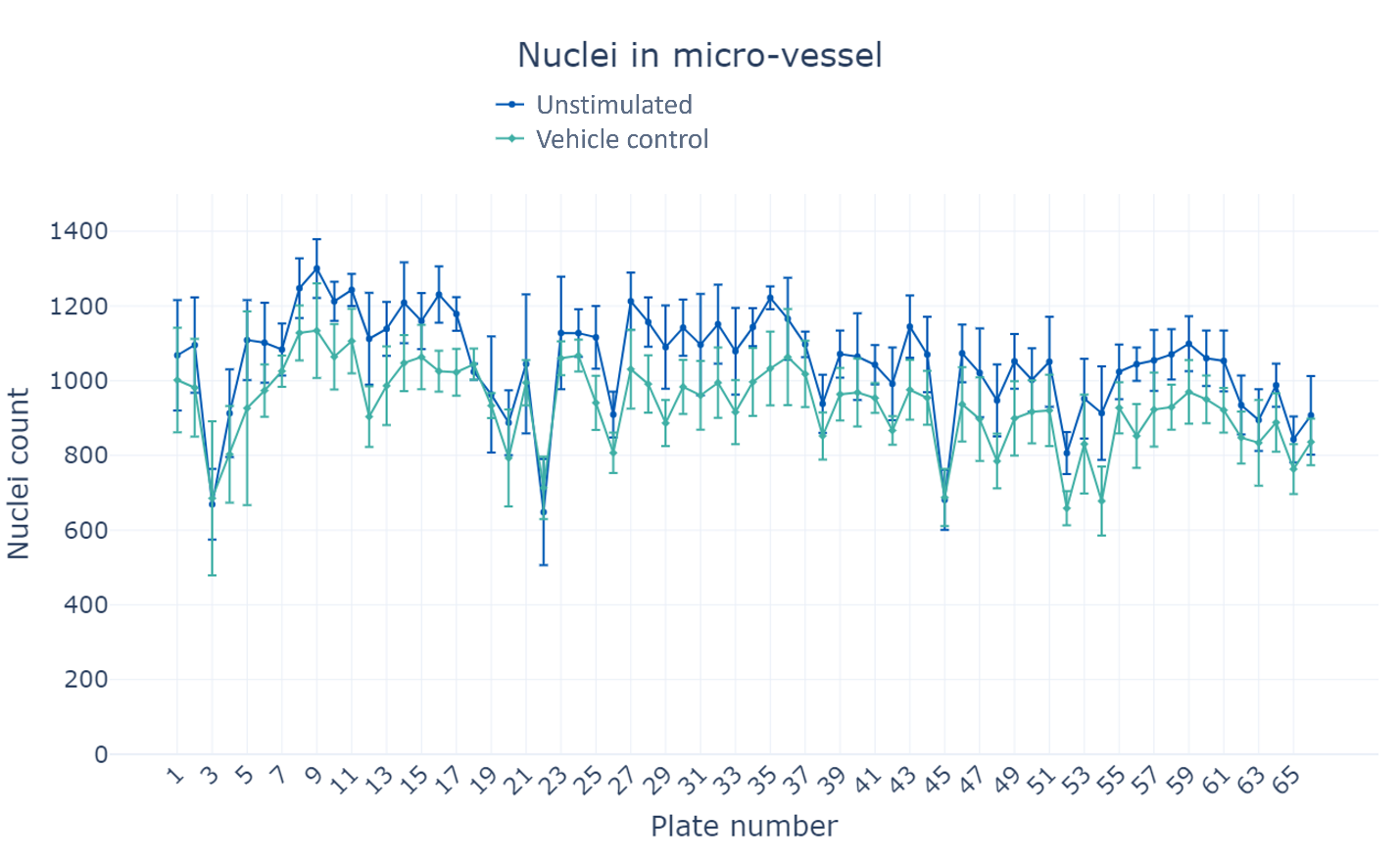


Fig. S 3 Nuclei count in the micro-vessel of unstimulated and vehicle control chips used in the screening. Each data point represented a plate and data were expressed as average ± standard deviation (AVG ± STD).


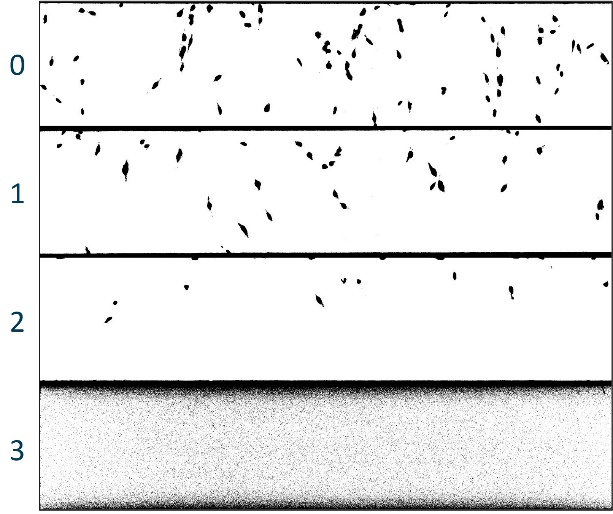


Fig. S 4 Representative binary images of inhibition levels (ILs) based on Z* scores with no (Z* scores above -3), mild (-3 to -9), moderate (-9 to -15), and high (below -15) levels of inhibition.


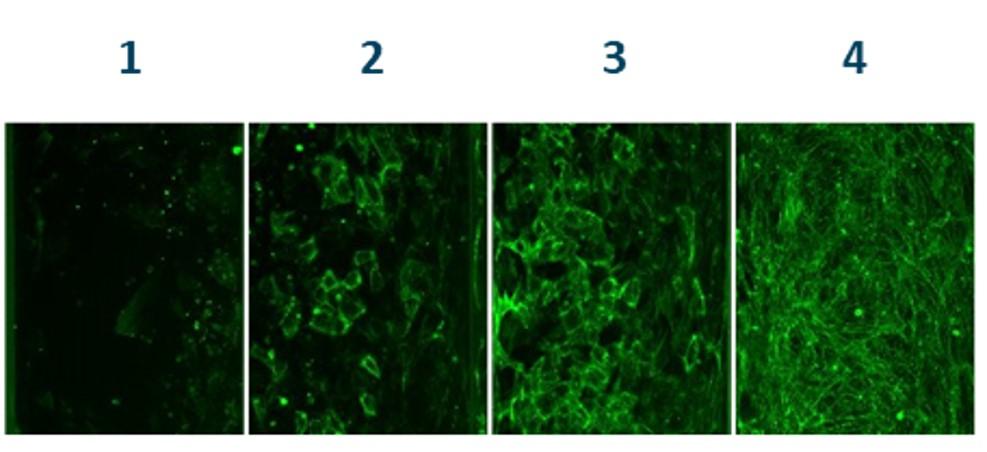


Fig. S 5 Representative images of the integrity scores evaluated by two experts in the micro-vessel. Scores ranged from 1 to 4 which indicated the level of toxicity where 1 = major toxicity, 2 =medium toxicity, 3 =minor toxicity, 4 = no toxicity. These scores (3 and 4) were used in combination with the Z* scores (<-3) to select hits.


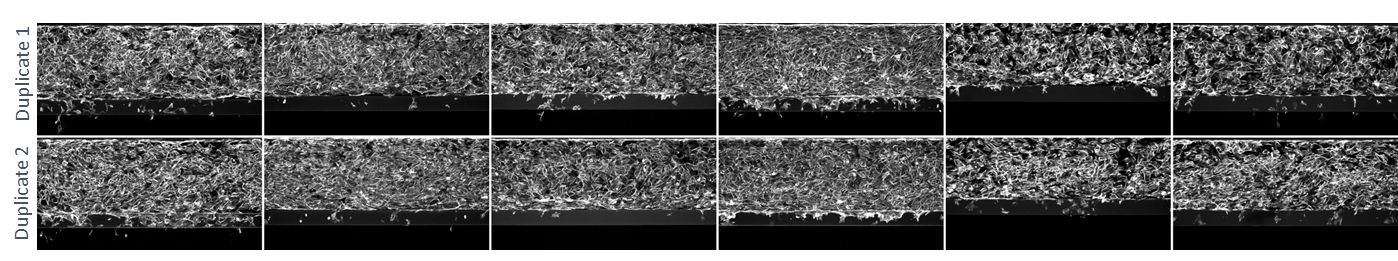


Fig. S 6 Greyscale images of F-actin staining of hits which targeted the MAP/ERK pathway.


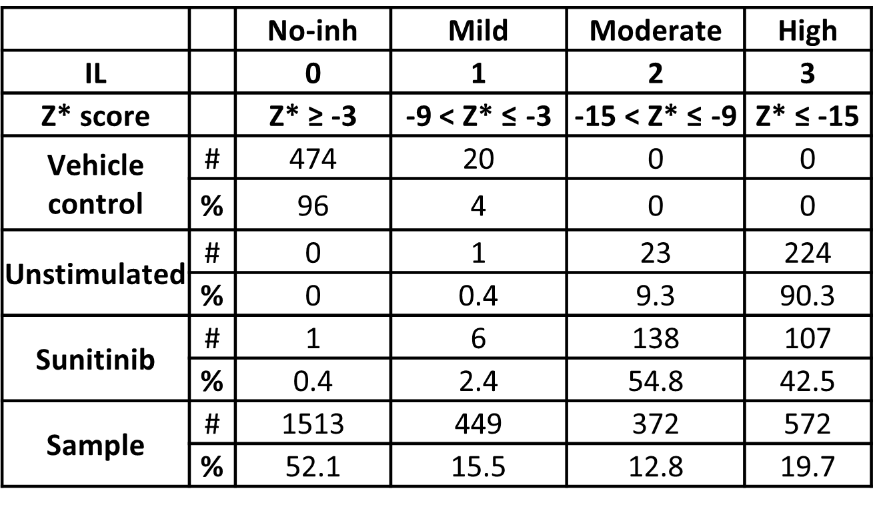


Table S 1 Number and percentage of hits per inhibition level (IL).

Fig. S 7. Fold change in nuclei size, aspect ratios (elongation) and shape complexity (irregularity – Perimeter2/Size) of the top 2 hits (MIM01 and MIM02) which targeted the MAP/ERK pathway in comparison to median of vehicle control nuclei. Nuclei morphometric information are extracted using ImageJ. Only concentrations with more than 10 nuclei counts are analyzed. Krustal Wallis non-parametric test with Dunn’s multiple comparisons performed for each concentration in comparison to the vehicle control * P<0.05, ** P<0.01, *** P<0.001, *** P<0.0001, otherwise non-significant.
